# Neural oscillations in the temporal pole for a temporally congruent audio-visual speech detection task

**DOI:** 10.1038/srep37973

**Published:** 2016-11-29

**Authors:** Takefumi Ohki, Atsuko Gunji, Yuichi Takei, Hidetoshi Takahashi, Yuu Kaneko, Yosuke Kita, Naruhito Hironaga, Shozo Tobimatsu, Yoko Kamio, Takashi Hanakawa, Masumi Inagaki, Kazuo Hiraki

**Affiliations:** 1 Graduate School of Arts and Sciences, The University of Tokyo, Tokyo, Japan; 2 Department of Developmental Disorders, National Institute of Mental Health, National Center of Neurology and Psychiatry, Tokyo, Japan; 3 College of Educational and Human Science, Yokohama National University, Kanagawa, Japan; 4 Department of Advanced Neuroimaging, Integrative Brain Imaging Center, National Center of Neurology and Psychiatry, Tokyo, Japan; 5 Department of Psychiatry and Neuroscience, Gunma University Graduate School of Medicine, Gunma, Japan; 6 Department of Child and Adolescent Mental Health, National Center of Neurology and Psychiatry, Tokyo, Japan; 7 Department of Neurosurgery, National Center Hospital, National Center of Neurology and Psychiatry, Tokyo, Japan; 8 Department of Clinical Neurophysiology, Neurological Institute, Faculty of Medicine, Graduate School of Medical Sciences, Kyushu University, Fukuoka, Japan

## Abstract

Though recent studies have elucidated the earliest mechanisms of processing in multisensory integration, our understanding of how multisensory integration of more sustained and complicated stimuli is implemented in higher-level association cortices is lacking. In this study, we used magnetoencephalography (MEG) to determine how neural oscillations alter local and global connectivity during multisensory integration processing. We acquired MEG data from 15 healthy volunteers performing an audio-visual speech matching task. We selected regions of interest (ROIs) using whole brain time-frequency analyses (power spectrum density and wavelet transform), then applied phase amplitude coupling (PAC) and imaginary coherence measurements to them. We identified prominent delta band power in the temporal pole (TP), and a remarkable PAC between delta band phase and beta band amplitude. Furthermore, imaginary coherence analysis demonstrated that the temporal pole and well-known multisensory areas (e.g., posterior parietal cortex and post-central areas) are coordinated through delta-phase coherence. Thus, our results suggest that modulation of connectivity within the local network, and of that between the local and global network, is important for audio-visual speech integration. In short, these neural oscillatory mechanisms within and between higher-level association cortices provide new insights into the brain mechanism underlying audio-visual integration.

The human brain is the primary organ responsible for our ability to function in, and adapt to, our environments. It accepts inputs through its many receptors, representing five primary sensory modalities, and processes the sensory input into appropriate motor output – one of the most fundamental abilities of human life. While sensory input can be processed one modality at a time (unisensory processing), the brain’s ability to process several modalities at once is also a critical component. Multisensory processing is the ability to combine cues from diverse modalities, and is a key brain function^1^ that is independent of intention, and directly contributes to perception. Findings of canonical studies of visual processing^2^,^3^ suggested that multisensory binding processes (i.e., processing the relationships between modalities) occur primarily after unisensory processing. Neurons that respond to stimuli from multiple modalities are located in the association cortices, such as the posterior parietal cortex (PPC), the inferior prefrontal cortex, and the superior temporal sulcus (STS)^4^.

Despite these findings, recent studies demonstrated that multisensory processing starts much earlier, namely during unisensory processing^5^,^6^,^7^,^8^. For instance, lip movements (visual stimuli) can activate secondary auditory areas very soon (10 ms) after the activation of the visual motion area, which causes a “phase-reset” that enhances the detection of the stimuli^9^. These studies demonstrated the early influence of multisensory processing in primary cortices^1^,^10^, but the mechanisms through which multisensory integration in higher-order brain regions takes place are still unclear.

Of the cross-modal brain regions like the STS and PPC, we have a special interest in the temporal pole (TP), also known as the anterior temporal lobe, which was recently hypothesized to be a convergent hub region capable of higher-level visual and auditory integration, including sematic processing^11^,^12^,^13^,^14^,^15^,^16^. For instance, the TP is assumed to play a more significant role than the STS in identifying a speaker as a representative higher integrative process^17^,^18^. To elucidate such functional aspects of the TP, we focused on “the cocktail party effect”, in which a listener isolates and attends a specific speaker despite external noises with the aid of visual cues such as the speaker’s mouth movements^19^,^20^,^21^. Hence, the cocktail party effect represents integration of audio and visual cues to enhance audition specifically^22^. We assumed that applying this effect would reveal the integrative processing of audio-visual information in the TP. Furthermore, one widely known aspect of such an integrative process that must be accounted for is the strong effect temporal factors have on multisensory integration^23^,^24^. For instance, cross-modal stimuli can be bound even when the two events occur at slightly different times, such that they are bound (perceived as a single event) even when occurring approximately 200–400 ms apart^25^,^26^,^27^. To account for temporal influences, we compared multisensory brain function between temporally congruent and incongruent audio-visual processing.

To describe the neural mechanisms underlying binding of speech-related audio and visual cues in the TP, we turned to analysing neural oscillatory activity, a prime candidate for explaining these systems^28^. Rhythmic brain activity supports the packaging and segmentation of information, and its coordination across distant brain areas. More recently, brain rhythms at different frequency bands were found to interact with each other^29^,^30^, a process called “cross-frequency coupling” (CFC) that has attracted increasing interest. One subtype of CFC, phase-amplitude coupling (PAC), has been particularly noteworthy. In PAC, low-frequency bands such as delta and theta modulate the amplitude of high-frequency bands such as alpha, beta, and gamma. PAC is assumed to represent an integrative process across neuronal populations and is a fundamental mechanism for information encoding^31^,^32^. Thus, this population-based effect describes local network encoding. For whole-brain network encoding, interregional oscillatory coordination (coherence) is thought to mediate information transfer across neural networks^33^,^34^,^35^. In fact, it appears to be crucial for perceptual and cognitive processes^36^,^37^,^38^, and particularly for cross-modal sensory processing^39^,^40^.

## Results

**Figure 1:**
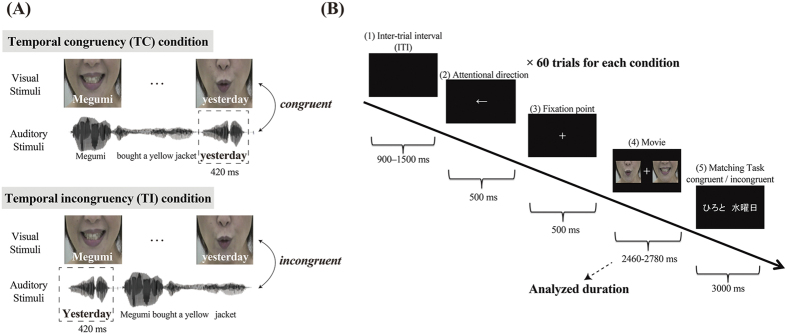
Experimental design. (**A**), Illustration of the two experimental conditions. In the temporally congruent (TC) condition, the audio and visual components of the movie were temporally congruent. In the temporally incongruent (TI) condition, audio-visual information was temporally incongruent (by 420 ms), e.g., a participant heard an audio segment such as ‘Megumi’ while watching a mouth movement such as “Yesterday”. In each trial, two stimuli were presented simultaneously, and the trial order was randomized throughout the experiment. (**B**), Experimental procedure. (1) Inter-trial interval (ITI) was 900–1500 ms, (2) a participant saw an indicator (<- or ->), which denoted the movie they should attend to. (3) the Fixation point was presented for 500 ms, (4) the movie (the TC or the TI condition) was presented for 2460–2780 ms. (5) A pair of matching or mismatching words was presented on the screen for 3 s. We analysed the signal in the last 500 ms of ITI for baseline in wavelet analysis, and used the movie section for source amplitude, power spectrum density, wavelet analysis, cross-frequency coupling, and imaginary coherence in the TC and TI conditions. It must be noted that our movies consisted of the time segment from 2460 to 2780 ms. Therefore, we used the time segment of 2400 ms during the movie segment, which was just before the movie ended.

In this study, we used magnetoencephalography (MEG) to determine how neural oscillations alter local and global connectivity during an audio-visual speech detection task (see Fig. 1). We selected regions of interest (ROIs) using whole brain time-frequency analyses (power spectrum density and wavelet transform), then applied PAC and imaginary coherence measurements to them to analyse the underlying oscillatory integration mechanisms.

### Behavioural results

**Figure 2:**
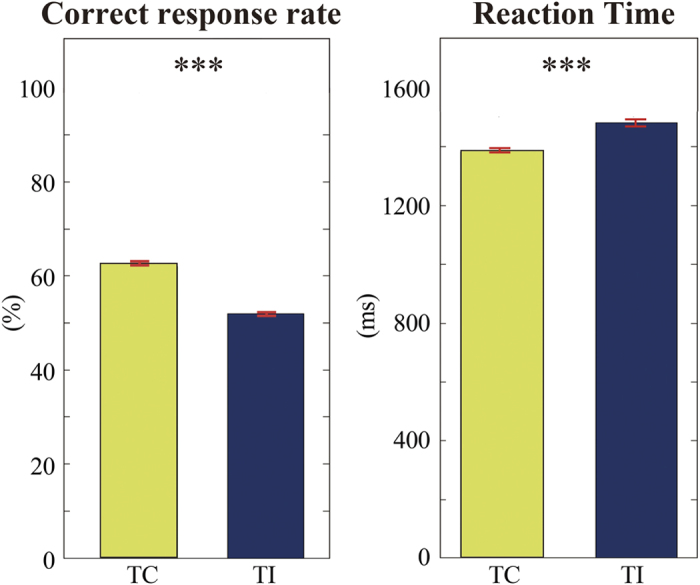
Behavioural Results. Correct responses and reaction times were averaged across participants. In the left panel, mean correct response rate (%) and 95% confidence intervals (the red bar) are shown. In the right figure, mean reaction time (ms) and standard error (the red bar) are shown. The significant difference in both accuracy and reaction time indicates audio-visual binding in the temporally congruent (TC) condition was conducted more successfully than in the temporally incongruent (TI) condition. *** P < 0.001.

We found that accuracy in the temporal congruency (TC) condition was significantly higher than in the temporal incongruency (TI) condition, which indicated that the audio-visual binding process was conducted more successfully in the TC condition (z-value 4.215, p < 0.001). Furthermore, the likelihood ratio test demonstrated that reaction time (RT) followed different ex-Gauss distributions across temporal conditions, which suggests that RT between the TC and TI conditions was significantly different (p < 0.001). Together, these results suggest that the binding process was less successful in the TI condition (Fig. 2), and support the observation that temporally congruent stimuli provide cues that enhance sensory processing, whereas temporally incongruent audio-visual stimuli did not. Therefore, we conclude that a temporally congruent audio-visual event between auditory (speech) and visual (mouth movements) signals is important for detecting speech in a noisy environment.

### Source estimation and power spectrum density

**Figure 3:**
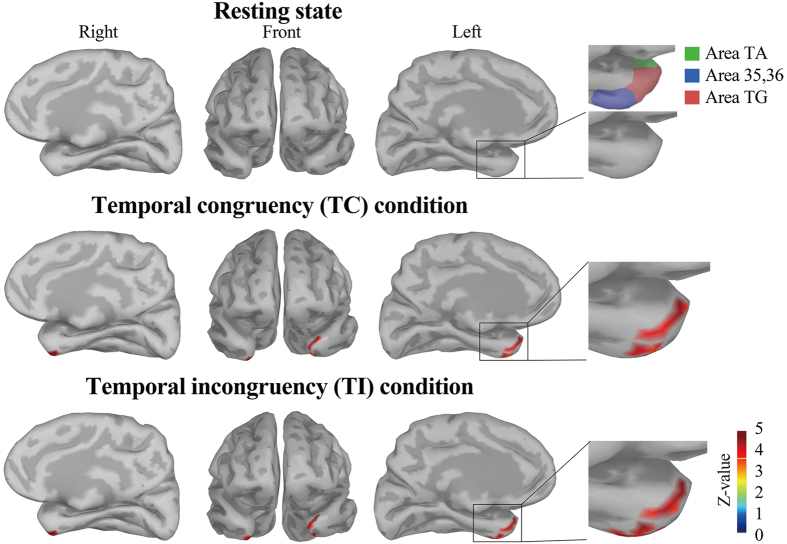
The spatial images of the delta power spectrum density z-score as three regions of interest (ROIs) in the bilateral temporal pole (TP) for the resting-state, temporally congruent (TC), and temporally incongruent (TI) conditions during the movie section. The anterior temporal lobe, especially the medial and ventral parts, was persistently activated by our task for both conditions. Red coloured areas in the TP denote the highest delta band PSD during the movie section in each condition. In addition, this figure contains the enlarged views of the subregions of the TP. For further detail, refer to the supporting material Fig. S1. Top - the resting-state condition; middle - the TC condition; bottom - the TI condition. In comparison to the resting-state data, the clusters in the TC and TI conditions showed significantly enhanced activities in the delta band (Bonferroni correction, p < 0.001).

To identify the main target region (ROIs) for the movie segment, we first calculated the group-averaged power spectrum density (PSD) z-score. Figure 3 illustrates the spatial images of PSD z-score as three parts (ROIs) in the bilateral TP for three experimental conditions. These areas consist of the subregions of the TP, namely the Area TG, the rostral part of TA (TAr) in the left TP, and the bilateral Area 35, 36. (The anatomical structures and currently accepted definitions of the subregions of the TP are provided in supplementary Fig. S1) Based on our threshold (i.e., below p < 0.0001), only three clusters implemented with delta oscillations were detected; in the TC condition, one ROI across the left TG and Area TAr (the upper parts [7 vertices, 1.93 cm^2^], the lower ROI in the left Area 35, 36 [the 12 vertices 3.25 cm^2^]), and the other ROI in the right Area 35, 36 (12 vertices, 2.42 cm^2^). In the TI condition, one ROI across the left TG and Area TAr (the upper parts [9 vertices, 2.45 cm^2^], the lower ROI in the left Area 35, 36 [the 10 vertices 2.74 cm^2^]), and the other ROI in the right Area 35, 36 (9 vertices, 2.12 cm^2^). Although the power spectrum density measurements demonstrated statistically significant brain activations mainly within the TP, several other brain regions including the PPC and occipital areas showed frequency-specific activities consisting of alpha and beta bands. However, the PSD z-scores of alpha and beta bands in the PPC and occipital areas were relatively small, and did not reach our p-value threshold (i.e., did not reach statistical significance).

In comparison to the resting-state data, we found that all of the ROIs demonstrated enhanced delta bands activities, which indicated that the delta bands activities in the ROIs were task-related, and significantly modulated by our task (the ROI in the left TG and Area TAr: the TC vs. rest, t = 7.5153, p < 0.001, the TI vs. rest, t = 10.4087, p < 0.001; the left lower ROI in the Area 35, 36: the TC vs. rest, t = 12.2619, p < 0.001, the TI vs. rest, t = 12.479, p < 0.001; in the right Area 35, 36: the TC vs. rest: t = 8.6856, p < 0.001, the TI vs. rest: t = 3.6838, p = 0.002). On the other hand, we did not find a significant difference between the TC and the TI in three ROIs (t = −0.0207, p > 0.05).

### Morlet wavelet transform in the temporal pole

**Figure 4:**
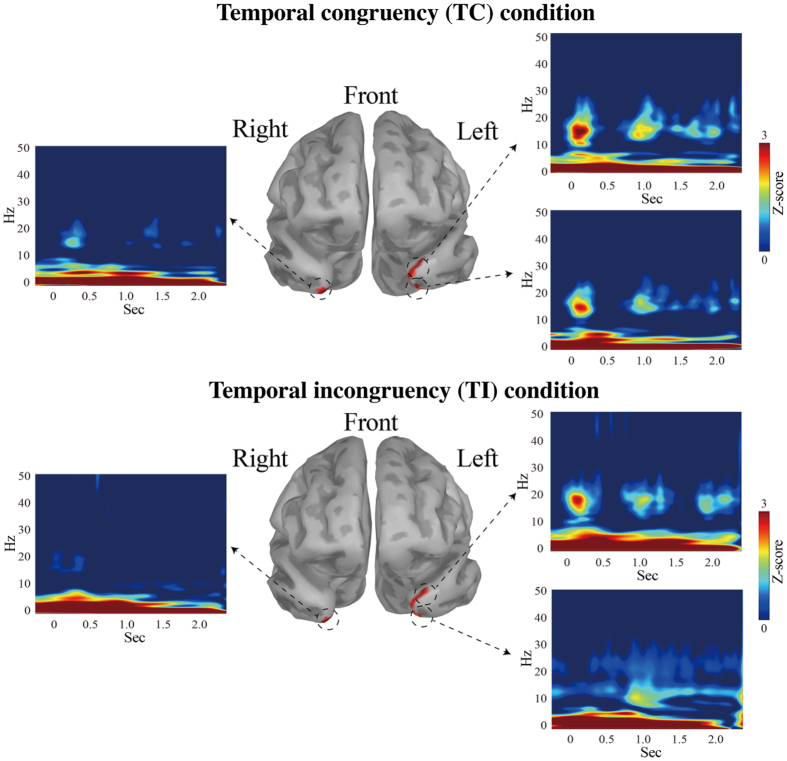
Z-score of wavelet transform of source data from three regions of interest (ROIs) in the temporal pole (TP). This figure shows that the multiple frequency components were observed in the clusters in the left hemisphere, but not in the right. Although multiple components were observed, the strongest power was detected in the delta and beta bands. The horizontal line denotes time sequence, and the vertical line indicates frequency (Hz). The activities of the delta bands were significantly enhanced only in the movie section, and not in the preparatory periods (Bonferroni correction, p < 0.001). Upper figure; the temporally congruent (TC) condition, Lower figure; the temporally incongruent (TI) condition.

Figure 4 demonstrates the transition of the power spectrum changes in time and frequency domain in the ROIs. As shown in Fig. 4, multiple frequency components were revealed. Among three ROIs, higher power spectrum in Area TG and TAr in the left hemisphere for delta (3–5 Hz), theta (6–8 Hz), alpha (9–12 Hz), and beta (13–30 Hz) were evident in both conditions. As seen, the strong sustained power pattern in delta was continuously observed for both conditions, and the activities of delta bands only were significantly enhanced in the movie section (delta_movie_ vs. delta_preparatory periods_, t = 5.1451, p < 0.001). In contrast, we did not observe such a specific response to the movie in the other frequency bands (see supplementary Fig. S2). Therefore, we concluded that the delta oscillations might be mostly task-related brain activities, and play an important role in binding audio-visual information. In addition, beta oscillations were periodically observed as a cluster, especially in the left hemisphere. However, the clusters of beta oscillations were detected not only in the movie section, but also in the attentional section (Fig. S2). Theta and alpha showed relatively weak power patterns, compared with beta and delta. These patterns were similarly observed in both experimental conditions. An analysis of variance (ANOVA) with 3 factors (ROIs, frequency, and the experimental condition) did not reveal any significant factors (ROIs (F = 0.71, p = 0.4906), frequency (F = 0.43, p = 0.827), and the experimental conditions (F = 0.16, p = 0.6938). In summary, these results suggest that the delta oscillations of the TP might be task-related brain activities, but the oscillatory activities in the TP cannot fully explain the difference between our experimental conditions, i.e., audio-visual temporal congruency vs. incongruency.

### Phase-amplitude coupling in the temporal pole

**Figure 5:**
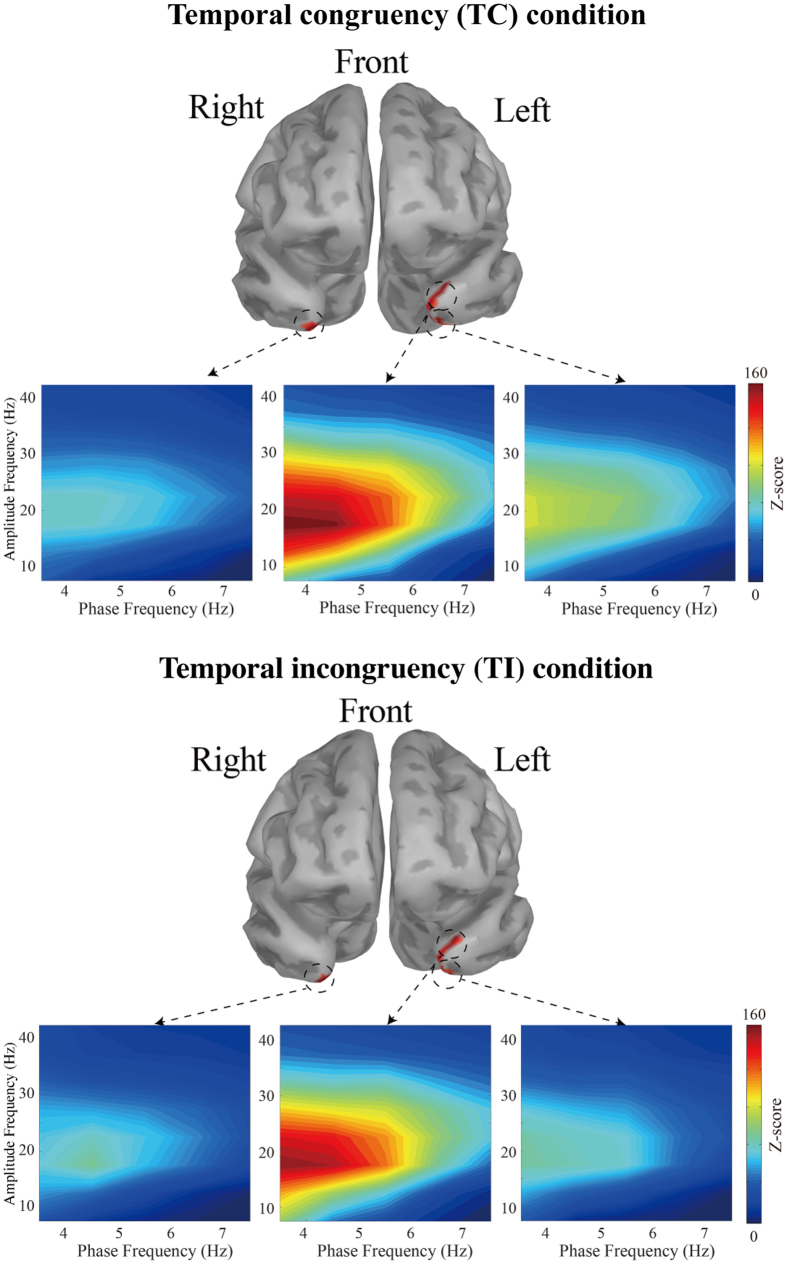
Phase-amplitude comodulograms for each condition. Vertical axes denote amplitude, while horizontal axes denote phase. Upper images show the phase-amplitude coupling (PAC) z-score for the temporally congruent (TC) condition, and lower images show the temporally incongruent (TI) condition for each region of interest (ROI). The 7 vertices in the left upper part of the temporal pole (TP) showed the strongest coupling, compared with the other ROIs in the left ventral part and the right TP. The vertices in the left upper part of the TP overlap the medial part of Area TG and the Area TAr. The left upper vertices demonstrated a significantly higher nesting among ROIs (Bonferroni correction, p < 0.001).

We observed multiple frequency patterns, especially in the left ROI, namely Areas TG and TAr. To determine the form(s) of CFC in these bands, we used comodulograms to reveal a novel phase-amplitude coupling pattern between 3–5 Hz (nesting frequency: delta) and 13–30 Hz (nested frequency: beta), compared to the areas in the right hemisphere (Fig. 5, in the TC condition, left upper vertices vs. left lower vertices, t = 4.0541, p < 0.001; left upper vertices vs. right vertices; t = 6.4611, p < 0.001; in the TI condition, left upper vertices vs. left lower vertices, t = 5.4225, p < 0.001; left upper vertices vs. right lower vertices, t = 5.8865, p < 0.001). As mentioned above, PAC can be a neural mechanism of binding audio-visual information from different sensory areas. Taking these statistical results into account, we marked the upper vertices in the left TG and TAr as the seed regions for binding audio-visual information. We therefore chose the left TG and TAr as the seed region for the next analysis. It must be noted that the PAC z-scores observed in these three areas were not significantly different between the TC and the TI conditions.

### Long-range imaginary coherence analysis

**Figure 6:**
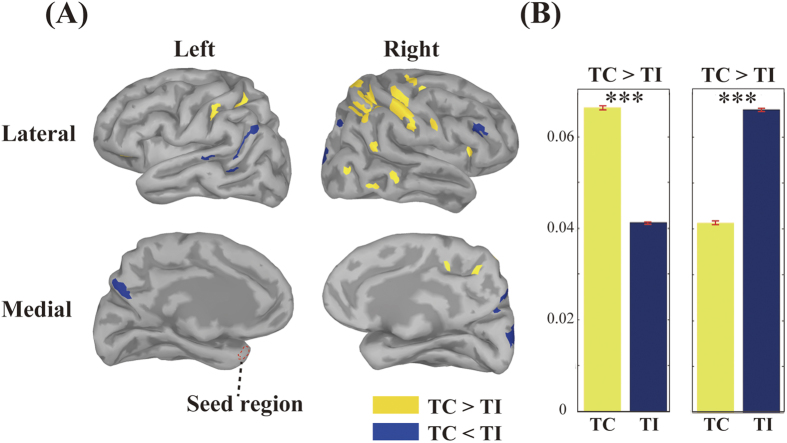
Imaginary coherence for each condition. (**A**), The yellow colour indicates clusters that are significantly higher in the temporally congruent (TC) condition than the temporally incongruent (TI) condition. The blue colour shows clusters significantly higher in the TI condition than the TC. To create this figure, coherence values in each time window were averaged across multiple time windows to elucidate sustained long-range networks during the movie section. Significant TC clusters tended to overlap with the posterior parietal cortex, including the post-central areas bilaterally. On the other hand, significant TI clusters tended to converge in the occipital lobe, the superior temporal sulcus, and the right ventral lateral prefrontal cortex. Lateral views are shown at the top, medial views at the bottom. (**B**), The averaged coherence values. The left and right figures indicate the average of the significant clusters detected in the analysis. The left yellow bar denotes the averaged imaginary coherence value plotted in yellow in Fig. 6A, whereas the blue bar in the right figure indicates the mean value of the blue clusters mapped in the same figure. Each red bar indicates standard error. ***Bonferroni correction P < 0.001.

After choosing the seed region, long-range imaginary coherence analysis was performed to estimate the brain regions differentiated by the experimental conditions. Using the seed region (the left ROI), we conducted an imaginary coherence analysis, and then compared imaginary coherence values on each vertex between the conditions using the Wilcoxon rank-sum test. Figure 6 demonstrates imaginary coherence maps based on the significant clusters between conditions, based on two statistical estimations (TC > TI and TC < TI) in the delta band. These significant clusters were observed as the delta imaginary coherence, and only the delta imaginary coherence was significantly modulated by our experimental conditions. Significant areas in the TC > TI condition (yellow) were clearly seen in the right lateral view (Fig. 6A right upper, z-value: 12.1524, p < 0.001). These clusters were observed in the parietal lobe, including the motor areas. On the other hand, significant clusters were also observed in the TI condition and are denoted by the blue colour (Fig. 6A middle, z-value: 4.739, p < 0.001). These clusters converged in the occipital lobe, the STS, and ventrolateral prefrontal cortex (VLPFC). Figure 6B indicates the statistical results of the Wilcoxon rank-sum test. Thus, these results suggest that in the TC condition, audio-visual binding depends on the functional connectivity among the TP, the PPC, and the motor-related areas; whereas in the TI condition, it relies on the functional connectivity among the TP, STS, VLPFC and the primary visual regions to detect temporal incongruency.

## Discussion

In this study, neural cross-modal mechanisms for binding audio-visual speech information were investigated. We specifically focused on sustained local and long-range neural computations in the TP, recently identified as a putative hub of multisensory integration activity. First, our behavioural data indicated that cross-modal stimuli must be temporally congruent for integration to be successful (Fig. 2). At the local level, our study revealed that delta oscillations in the TP were related to our audio-visual sensory integration task (Fig. 3). To the best of our knowledge, this is the first study to use CFC to reveal a novel delta phase–beta amplitude coupling partially localized to the left TP, and specifically to the medial part of Area TG and TAr (Fig. 5). At the global (long-range) level, network analysis demonstrated delta band coherence between the TP and the PPC, including the motor areas important in audio-visual speech processing (Fig. 6). Below, we focus on two key points related to our findings: The functional role of PAC in the TP, and the role of global network functional connectivity for binding audio-visual modalities in speech processing.

Previous studies have demonstrated perceptual units of analysis of audio-visual speech are composed across a range of time scales^41^,^42^. For instance, the acoustic envelope of speech closely correlates with the syllable rate, such that short duration phonemes such as/ha/ are tightly related to the fine structure of speech. Similarly, visual components including mouth and head movements accompany acoustic speech as additional information; they are not only correlated with the syllable rate, but also the phrase boundary. Therefore, the temporally rhythmic information in speech might be relevant to its decoding. More importantly, these perceptual units also correspond well with neural oscillations of similar frequencies. That is, as our data suggest, the TP uses delta band oscillations during processing. In fact, the syllabic rate peaks at 4 and 7 Hz (delta/theta), and the augmentation of speech signals into lexical and phrasal units (e.g., intonation) occurs at approximately 1–2 Hz. Therefore, Giraud and Poeppel have proposed that “the brain converts speech rhythms into linguistic segments” using neuronal oscillations^43^. According to their hypothesis, the low gamma, high gamma, delta, and theta bands might correspond to various language components. In fact, a number of studies have reported that neural oscillations underlie speech information coding^38^,^44^. Thus, neuronal oscillations appear to function as encoders, turning speech signals into neural information units.

Notably, several reports indicate that the above neuronal oscillatory mechanism can be applied to speech-related signals from both audio and visual modalities^45^,^46^. Specifically, low frequency (delta and theta band) oscillations can encode and carry dynamic audio-visual speech information. Luo *et al.*^39^ demonstrated that low frequency bands (delta and theta bands) might represent a synergistic coordination of audio-visual information. It should be noted that Ding and Simon demonstrated that delta oscillations play a more significant role than theta oscillations during the processing of attended speech, and that delta band activity is more accurate than theta for decoding speech^47^. As seen in Figs 3,4 and 6, our data are consistent with these other studies: Here, audio-visual speech stimuli modulated delta band oscillations in the TP. Therefore, we conclude that the delta oscillations in the TP induced by audio-visual stimuli represent neural oscillatory activity related to audio-visual speech processing.

Given the above roles of oscillatory activity, the question remains of how temporally congruent individual sensory components (audio and visual) are combined to encode (or enhance) the perceived speech. One candidate computational mechanism is the interaction of low and high frequency band oscillations. The best-known interaction of this type is the aforementioned phase-amplitude coupling, in which a low frequency phase is correlated with a high frequency amplitude.

PAC was first observed in subcortical areas, especially the hippocampus, and significant progress in the field has been made in rodent brains through analysis of place cells. In place cells, PAC between theta and gamma bands represents the sequential position of an animal in space^48^,^49^,^50^,^51^. In a one-dimensional task, an individual assembly of place cells fires (usually at gamma frequency) maximally at particular positions. This firing is essentially coupled to the trough of the theta phase, such that multiple cell assemblies are sequentially co-activated in any given theta cycle; thus, information about successive locations and distances is packaged without losing the precise temporal sequence within a theta cycle^32^. Based on these findings, PAC observed in human studies is similarly assumed to be a mechanism of packaging sequential information. Generally, it is thought that high frequency brain activity represents local computations (akin to place cells), while low frequency delta/theta activity is entrained across the global brain network. This framework is a good fit for data from previous studies^31^. In fact, one very recent study using electrocorticographic recordings elegantly demonstrated that TP oscillations in voice- or face-naming tasks were characterized by beta band activity^11^. Importantly, in our data as seen in Fig. 5, the beta band activity was coupled with the task-related delta band activity, which supports the idea that beta oscillations in the TP were related to processing combined audio-visual information. Taken together, the data in our study suggest audio-visual speech information might be sequentially packed in the TP via PAC.

Functional connectivity in long-range networks is measured by the degree to which oscillatory activity in two potentially connected areas is temporally correlated. Strongly connected regions have oscillatory activity that is closely correlated in time. One way to measure this connectivity is via coherence. Coherence is the degree to which two signals maintain a fixed phase relationship with each other. Since coherence measures spectral covariance, it cannot accurately divide phase and amplitude contributions. However, previous studies have demonstrated that it works well for describing co-activation across the brain regions. Strong functional connectivity is believed to require good coherence, since inputs arriving at random phases will not integrate as effectively. Conversely, highly coherent inputs will integrate into a strong signal, enhancing communication (and functional connectivity) between areas^52^,^53^. For instance, Schroeder and Lakatos^53^ demonstrated that low frequency oscillations ensure that local neurons are in a high excitability (i.e., “up”) state, during which information processing is enhanced. Stimuli arriving out of phase with the up state (i.e., during the “down” state) are either ignored or suppressed. Thus, the phase of the low frequency oscillation is an “adaptive temporal filter” that can gate information flow between brain regions.

One difficulty when measuring coherence is “signal leakage” or “volume conduction”, which detects artefactual rather than true interactions. To counter this problem, we calculated imaginary coherence, which can apply spectral coherence without concern for false connectivity due to volume conduction. We then computed the imaginary coherence in terms of functional coordination between the TP and the other brain areas.

In our study, the imaginary coherence in the TI condition among the TP, STS, and VLPFC was significantly higher than in the TC condition as seen in Fig. 6. We believe the specific functions of the above areas explain this finding. The STS is known to be sensitive to cross-modal object features, such as audio-visual information^4^. The VLPFC, which receives direct input from the STS, is believed to be a part of an auditory pathway processing “what” queries for complex sound features^54^,^55^. Therefore, it is possible that the temporal incongruence “stalled” a lower-level audio-visual integration process in the STS and VLPFC, and thus the higher-level association cortex (TP) was required for processing. Support for this is found in the functional difference between the STS and the TP in terms of multisensory integration. For instance, relative to the TP, the neural responses of the STS are more sensitive to conspecific voices and dynamic faces, but much less so to auditory-vocal features such as individual identity^16^,^17^. In our task, participants were required to identify the target utterance in a cocktail party situation, which meant picking an individual utterance, and specific speech/semantic segments, out of a pair of voices. This requirement would thus necessitate coordination with the TP^56^,^57^.

On the other hand, functional connectivity in the TC condition was significantly enhanced between the TP, the PPC, and the post-central area. The PPC is traditionally regarded as an important brain region for multisensory perception, and PPC neurons that respond to cross-modal stimuli have been identified^58^. Similarly, anatomical connectivity indicates that this brain area is a convergent hub of auditory, somatosensory, and visual motion systems^59^. Indeed, applying anodal transcranial direct current stimulation over the right PPC prevented the multisensory integration of visual and auditory features^60^,^61^. Furthermore, clinical reports have demonstrated that PPC lesions cause feature-binding deficits^62^. Thus, our imaginary coherence analysis indicates that with temporally congruent, cross-modal speech information, the long-range network for integrating such signals is comprised of multiple audio-visual or tri-sensory association cortices^63^.

Accuracy and RT were both significantly different between the TC and the TI conditions. This behavioral analysis unambiguously demonstrated the audio-visual incongruency effects in the present task. However, the interpretation of the neurophysiological measures requires caution, since in addition to the incongruency effects, the difference in task difficulty in general can influence the results of imaginary coherence. For instance, increased task difficulty in general may induce additional process such (e.g., increased attention demand), and this factor could increase functional connectivity in the STS and VLPFC (Fig. 6). Although the present experimental design could not completely separate the temporal incongruency effects from the task difficulty effects, task difficulty alone unlikely explained the imaginary coherence findings for the following reasons.

There is considerable evidence supporting the roles of the STS and VLPFC in the integration of audio-visual speech^14^,^17^,^63^. In particular, a previous study demonstrated that activations in the STS in response to incongruent information between face-voice stimuli cannot be explained solely by task difficulty. Rather, the author determined that activations in the STS were due to detection of mismatching information between the visual and auditory information^64^. Therefore, we believe that the enhanced activations in the TI condition in this study primary reflected the integration of audio-visual information more than task difficulty.

Anatomical connectivity studies of the TP provide support for our imaginary coherence data. Diffusion tensor imaging studies revealed that anatomical connections of the TP are intra-regionally distinct^13^,^65^. Specifically, the TAr regions (see supplementary information and Fig. S1) have a connection to the association auditory cortex in the superior temporal gyrus, which implies that the TAr is part of the auditory “what” pathway. Furthermore, the TAr strongly connects to the inferior frontal gyrus, a part of the VLPFC, via the uncinate fasciculus. Additionally, the inferior longitudinal fasciculus, which was reported to convey visual information from the occipital lobe to the temporal lobe, can be distinguished in the TG, a subregion of the TP^13^. Finally, Binney and colleagues reported that the arcuate fasciculus extends from the TP to the parietal language areas^65^. The data on anatomical and functional connectivity thus suggest that multisensory processing, at least for audio-visual stimuli, requires several cortical areas to work in concert with each other, to detect the presence of temporal incongruence in cross-modal stimuli; and when the stimuli are temporally congruent, to achieve high-level multisensory congruent perception.

Multi-sensory processing was investigated using MEG to assess activity in an audio-visual speech task. By integrating minimum-norm estimate (MNE), PSD, wavelet, PAC, and imaginary coherence analyses, we identified both local and global constituents of a network were involved for integration of audio-visual speech information. We also elucidated the underlying oscillatory mechanisms of information encoding in one local area (the TP) and in the larger network (inter-regional coherence). Specifically, we identified the temporal pole as a key network component, and delta band activity in it as necessary for encoding audio-visual stimuli. The left TP demonstrated coupling between the delta phase and the beta amplitude indicative of sequential information coding. In terms of functional connectivity and global network activity, the TP was a key component in long-range networks related to processing both temporally incongruent and temporally congruent multimodal stimuli. Our data indicate a mechanism through which the TP acts as a hub in the larger network. Delta band phase modulates both signal encoding (binding) in the TP, and information transfer between the TP and other network components. Integration of multisensory stimuli is an important aspect of the socially critical task of perceiving speech in a noisy environment, and our data provide novel insights into the mechanisms through which the brain accomplishes this.

## Limitations

In this study, we evaluated audio-visual speech matching without addressing the effect of spatial deployment of attention. That is, we treated the hemispheres of the brain equally in terms of attention, and did not analyse the attend-to-left trials and the attend-to-right trials separately. Although we observed the strongest coupling in the left TP, it is widely known that attending to the right visual fields induces greater neural activity in the left hemisphere. Therefore, the lateralization of PAC seen in Fig. 5 might be caused by the differential effect of spatial attention deployment between hemispheres. However, our experiment was not designed to include trigger signals that would allow us to distinguish attend-to-left trials from attend-to-right trials, and therefore we cannot exclude such a possibility. A future study is expected to investigate whether such hemispheric dominance-related effects are observed in the TP.

## Materials and Methods

### Participants

Nineteen native Japanese-speaking participants (fifteen men, mean age 25.6 years, all right-handed) with normal hearing, normal vision, and no history of neurological, psychiatric, or developmental disorders were enrolled in our study. All participants gave informed written consent. All provided informed consent under a process and the experimental protocol approved by the National Center for Neurology and Psychiatry Research Ethics Committee, were carried out in accordance with the latest version of the Declaration of Helsinki. All participants were assessed with attention deficit/hyperactivity disorder and autism spectrum disorder scales, and with the Wechsler Adult Intelligence Scale 3 (WAIS-3), to allow for the exclusion of subjects with possible undiagnosed developmental or neuropsychiatric disorders. Two participants were excluded from our analysis due to excessive body movements during the experiment, and two participants were excluded due to inadequate WAIS-3 scores (i.e., less than 85 points in four indices of the WAIS-3). Data from the remaining fifteen participants were therefore analysed in our study.

### Materials and experimental design

We created stimuli consisting of 200 movie clips of a professional female announcer speaking. It is widely accepted that watching mouth movements enhances speech comprehension even in a noisy environment, so we focused on recording mouth movements. Each movie consisted of an emotionally neutral, 5-word sentence (2460–2780 ms long), such as the Japanese equivalent of “Megumi bought a yellow hat yesterday”. Words used in this experiment consisted of 3–4 morae in order to control the duration of the speech. For each subject, the task involved two movies presented simultaneously that contained different sentences, but utilized the same syntactic structure (subject, transitive verb, adjective, noun, and adverb). The movies were edited using Adobe Premiere Pro CS6 to position the face in the centre of the frame, equalize the relative size of the mouth, and clip the movies precisely. Audio signal levels (volumes) were measured as root-mean-square contrasts, and normalized using MATLAB R2014a (MATHWORKS Inc., Natick, MA). The auditory signals were presented through in-ear earphones (Etymotic ER3-A) such that the speech sounds were presented at a comfortable conversational level (sound pressure level (SPL) = 72 dB). The visual stimuli were projected through an opening in the wall onto a back-projection screen situated in front of the participants, who were inside a windowless shielded room. Before starting the experiment, participants were required to fix their gaze on a fixation point on the screen, and were asked whether they could clearly see the mouth movements in the movies using their peripheral vision. As a result, each stimulus was located at −11° horizontal and 10° vertical visual angles. Then, subjects were instructed to keep their eyes on the fixation point during the videos. All stimuli and triggers were controlled using Superlab 5 (Cedrus).

To account for temporal factors, we created the TC and the TI; the control condition. In the TC condition, visual (mouth movements) and auditory (speech) stimuli were temporally congruent, which enhanced speech detection. On the other hand, in the TI condition, speech and mouth movements were temporally incongruent (a 420 ms mismatch). This difference attenuates the binding process and the ability to understand speech. To create the TI movies, we analysed both the spectrogram and sound^66^ and, while leaving the visual element intact, clipped the last auditory segment, for instance the word “yesterday”. Then, the clipped auditory segment was transposed to the start of the sentence to produce the TI sound track, as in “Yesterday Megumi bought a yellow hat.” The duration of the clipped auditory part was approximately 420 ± 20 ms (Fig. 1A).

As a jitter, we included an inter-trial interval that varied randomly from 900–1500 ms for each trial. At the beginning of each trial (the preparatory period), instructions appeared in the centre of the screen for 500 ms, indicating whether participants should attend to the left or right, and the fixation point appeared for 500 ms (Fig. 1B). Then, the two movies were presented simultaneously. In Fig. 1B, the movie on the left side was the target, whereas the other movie on the right-hand side acted as a proximate noisy environment (i.e., a distractor). For a given temporal condition (TC/TI), both movies presented were of the same temporal congruency. After watching the instructions and movies, two words were written on the screen, and participants were told to indicate via button press whether the two words were contained in the sentence towards which they had directed their attention. For example, for the attended sentence: “Hiroto bought a yellow hat yesterday”, the words might be “Hiroto yesterday” (match/yes), or “Megumi yesterday” (mismatch/no). Sentences were not repeated across trials. It should be noted that attending to mouth movements was likely the only solution for comprehending the target speech, since both movies showed utterances by the same woman, and the two auditory streams corresponding to the two movies were presented simultaneously, and without lateralization, through the two earphones. Therefore, participants were instructed to attend to mouth movements before the experiment started.

### Behavioural analysis

Using a generalized linear mixed model, we analysed accuracy in the task. We used a binomial distribution for the accuracy data. Studies have reported that RT data fit an exponentially modified Gaussian (ex-Gauss) distribution^67^,^68^, so we first tested our RT data for such a fit. We subsequently conducted a likelihood ratio test to confirm whether the RT data derived from the TC and TI conditions specifically would follow identical ex-Gauss distributions.

### Magnetoencephalography and Magnetic Resonance Imaging (MRI) data collection

MEG data were recorded using a high-density whole-scalp VectorView MEG System (Elekta-Neuromag, Helsinki, Finland), containing a magnetometer and two orthogonal planar gradiometers at each of 102 positions (306 sensors in total). The experiment was conducted in a magnetically shielded room. We recorded two sets of MEG data for each participant: First, participants underwent a 4 min long, eyes-closed resting-state MEG recording session; then they were scanned during the trials. Data were sampled at 1,000 Hz with a bandpass filter of 0.03–330 Hz. Using a 3D digitizer (Fastrak, Polhemus), we recorded the positions of four head-position indicator (HPI) coils, the nasion, right and left pre-auricular points, and more than 100 additional points randomly dispersed over the scalp. After applying the HPI coils, the head position was continuously monitored, which allowed for movement compensation across the entire recording session. Two channels with excessive noise were marked as bad channels, and rejected.

It has been reported that saccadic movements cause noise in high frequency bands, especially in the temporal cortex. Therefore, controlling saccadic eye movements was necessary for our task and analyses. Electrocardiogram (ECG) and electro-oculogram (EOG) signals were recorded to detect trials containing heartbeats, vertical and horizontal eye movements, and blink artefacts. In addition, 10 min of data were recorded in a vacant room and used for ambient noise normalization. Structural MRI T1-weighted images were obtained for all participants using a 3 T MRI scanner (Siemens 3T Verio) with a 12-channel phased-array receiver coil. A 1 × 1 × 1 mm voxel size was acquired using a 3D magnetization-prepared rapid gradient echo sequence (repetition time = 1900 ms, echo time = 2.52 ms, flip angle = 9°, acceleration factor 3D = 1).

### Data analysis

To remove external noise and correct for head movements, the temporal extension of Signal-Space Separation^69^ was implemented off-line in MaxFilter 2.0 (Elekta-Neuromag), and a notch filter was applied to the data to remove interference from the 50-Hz alternating current and its harmonics (100, 150, 200, 250 Hz). All data analyses were performed using MATLAB, Brainstorm, and FreeSurfer^70^,^71^. As seen in Fig. 1B, the task-related MEG data were segmented into a single trial lasting 3900 ms, encompassing the “inter-trial interval” (500 ms), the “attentional direction” cues (500 ms), the “fixation point” (500 ms), and the “movies” (2400 ms). A total of 70 trials were collected for each of the two conditions. For analysis purposes, we defined “movie” in Fig. 1B as the analysed time course (0–2400 ms). Trials were rejected if the peak during the trial exceeded 50 μV, 80 μV, 1,000 fT, and 3,000 fT/cm in any of ECG, EOG, magnetometer, and gradiometer channels, respectively. Furthermore, data segments from the trials that were above the threshold of 2 standard deviations for EOG, and 4 standard deviations for ECG, were detected by Signal Space Projections, confirmed via visual inspection, and removed. These manipulations resulted in the exclusion of 1–8 trials for each condition per participant. To fairly address the data size, and maintain a constant signal-to-noise ratio across participants and conditions, we selected and analysed 60 trials per condition per participant. In cases with over 60 unrejected trials, we randomly chose 60 trials. The same set of trials was used for all analyses for each participant. For the resting-state MEG data, we divided the original data into 4 chunks consisting of 1 min for each subject. For each chunk of resting-state MEG data, the identical manipulation to remove artefacts mentioned above was conducted. As a result, 59 resting-state data points (59 min, 15 participants) were used for the following analysis. For each subject, FreeSurfer was used to create dense triangulation of cortical surface data based on T1-weighted image data, and these data were co-registered to a standard brain imageset (MNI305 (FsAverage), Montreal Neurological Institute), using a spherical representation of the cortex^72^. Then, Brainstorm was used to downsample these vertices to 15002 vertices, corresponding to a spacing of ~0.3 cm^2^ per vertex.

### Source estimation and power spectrum density

These analyses identified ROIs significantly modulated by the experimental task. First, the task-related and resting-state data were compared. Source reconstruction was conducted using the MNE for both data, which makes minimal assumptions about the generators of brain activity^73^. This method conducts optimum source estimation for analysing complicated or totally unknown sources within the general spatial resolution limits of MEG measurements. An inverse solution was obtained, based on the forward solution of the lead field matrix, which models the signal pattern generated by a set of electric dipoles located on the surface of the cortex. For this computation, the overlapping spheres model was used. This model is based on the estimation of a different sphere for each sensor, and can estimate a sphere that fits locally the shape of the head in the surroundings of each sensor. To compute noise normalization, a noise covariance matrix was created using empty room data recorded for 10 minutes before the experiment. MNE was applied for each individual data point, and estimated individual source activations were transformed into the standard brain.

Next, based on the source estimation data, we calculated PSD (i.e., Welch’s method) at the whole brain level for delta, theta, alpha, beta, and gamma bands. Welch’s method estimates the power of the frequency content of the signal by applying a Fast Fourier transform, which reduces noise from the time-to-frequency domain conversion. We set the window length to 500 ms, and the window overlap ratio to 50%. The calculated PSD values in each window were averaged across all time windows. For the task-related data, the time segment consisting of “movie” in Fig. 1B was used. These PSD values were normalized such that they had a mean of 0, and scaled to have a standard deviation of 1 (PSD z-score). We calculated the grand average PSD z-scores, and transformed these values into p-values. We first identified the most prominent task-related activations in the frequency bands across the whole brain. Then, we identified clusters in the bilateral TP with the largest PSD z-scores in the delta band. We set p < 0.0001 as the threshold for ROI definition to improve specificity. Figure 3 shows PSD z-scores for each condition masked by the p-value threshold. Other vertices at the other frequency bands were not significantly activated below the p-value threshold. These thresholded z-scores were then compared to the resting-state PSD z-scores via t-test, to confirm whether these three clusters demonstrated task-related activity. Bonferroni’s multiple comparisons correction was applied as necessary. Throughout this analysis, clusters consisting of less than 5 vertices were considered noise.

### Morlet wavelet transform in the temporal pole (TP)

The Morlet wavelet is one of the most commonly used frequency-analysis methods, since it has a Gaussian window shape both in time and frequency, maintains a sinusoidal underlying structure^74^, and generates easily interpretable results in time and frequency domains. After the PSD analysis identified task-related brain activities in the TP, which were marked as three ROIs, we conducted Morlet wavelet transform for MEG source-estimation data in the ROIs to reveal the transition of the power spectrum changes in the time and frequency domains. First, the source waveform was extracted from each marked ROI, and Morlet wavelet transformation was applied to each brain signal for the three ROIs. These transformed signals were then averaged first in each ROI, and subsequently across the all participants. We set the wavelet width at 3 to detect transient activity, and used a set of wavelets ranging from 1–50 Hz in steps of 1 Hz. These results were standardized as the z-score and then averaged for each condition. In transforming the z-score, we used the inter-trial intervals (Fig. 1B) as a baseline (500 ms). For the z-scores of each of the frequency bands in ROIs, we applied 3-factor ANOVAs using location (ROIs), frequency, and experimental condition factors. For a complementary analysis, the data (3400 ms) including the preparatory period as well as the “attentional direction” and “fixation point” times described in Fig. 1B were also analysed; this was done to confirm whether the brain activities during the preparatory periods at the three ROIs were consistent with the “movie” part. (For detailed results, see Fig. S2 and the Discussion section). Correction of multiple comparisons was assessed with Bonferroni’s procedure.

### Phase-amplitude coupling in the temporal pole

In the brain, phase-amplitude coupling (also called nesting) is exemplified by low-frequency bands (delta/theta) modulating the amplitude of high-frequency bands (alpha and higher). To quantify the Modulation Index (MI) that denotes the degree of coupling, we applied the mutual-information method proposed by Tort^75^,^76^ to the three previously-described ROIs. Briefly, we first created a phase bin for each 20 degrees, and assessed the amplitude modulation by phase using a normalized entropy measure for the vertices showing multiple frequency components. In our calculation, PAC was quantified for each single vertex, each trial, and each participant. We then computed the median value for PAC across all “movie” segments within a condition. For a trial, we set the frequency range for phase at 3–12 Hz in steps of 1 Hz, and the array amplitude at 10–150 Hz in steps of 10 Hz, which revealed PAC between the delta phase and beta amplitude. To clarify the more detailed correspondence between phase and amplitude frequencies, and reduce the amount of computation in creating surrogate data, we specifically focused on 3–7 Hz for phase in steps of 1 Hz, and 5–40 Hz for amplitude in steps of 5 Hz, and performed the calculations again. Surrogate control analyses are important for evaluating proper coupling, especially when assessing the MI of short time periods, since random fluctuations in the signal could cause artefactual coupling^77^. To this end, we randomly shuffled the phase time series of each trial until the original phase sequences became completely random. Then, 200 surrogate MI values were generated, from which we could infer the MI chance distribution. Based on the MI chance distribution, the original MI was then standardized^78^. Finally, we applied t-tests with Bonferroni’s correction to these z-score comparisons in the three ROIs.

### Long-range imaginary coherence analysis

Coherence analysis is a measurement of phase covariance, which is quantified as the cross-spectrum of two signals divided by the product of the two auto-spectra. One problem when measuring coherence is “signal leakage” or “volume conduction”, which detects artefacts rather than true interactions. This is a result of the activity of a single generator within the brain being detectable in many channels outside the head. To prevent this, one solution is the use of imaginary coherence^79^. We computed the imaginary coherence between the seed region in the left TP with the strongest PAC (see the Results, PAC subsection, for details) and every other vertex (14986 vertices) over the whole brain. Briefly, the waveforms of vertices in the seed region were averaged to yield a mean time course for the seed region. Imaginary coherence analysis was then conducted using a moving time window on single trials for 2400 ms in the “movie” in Fig. 1B. The results were averaged during this epoch within each frequency band from delta, theta, alpha, and beta, to obtain a mean coherence value for each participant, and then averaged across the participants. We mapped the mean imaginary coherence values over the cortex above the threshold (more than 35% of the peak level). In this calculation, we regarded small vertices less than 5 as noise. When conducting statistical analysis, we used the Wilcoxon rank-sum test for imaginary coherence comparisons, because the coherence value is always positive and does not follow a Gaussian distribution, and corrected it with Bonferroni’s correction for multiple comparisons.

## Additional Information

**How to cite this article**: Ohki, T. *et al.* Neural oscillations in the temporal pole for a temporally congruent audio-visual speech detection task. *Sci. Rep.* **6**, 37973; doi: 10.1038/srep37973 (2016).

**Publisher's note:** Springer Nature remains neutral with regard to jurisdictional claims in published maps and institutional affiliations.

## Supplementary Material

Supplementary Information
