# Supplementary material for: Neural oscillations in the temporal pole for a temporally congruent audio-visual speech detection task
Source: Sci Rep. 2016 Nov 29;6:37973. doi: 10.1038/srep37973 (PMC5126633; doi:10.1038/srep37973)
Supplement: Supplementary Information [file srep37973-s1.pdf]

Neural oscillations in the temporal pole for a temporally congruent audio-visual speech detection task

Takefumi Ohki, Atsuko Gunji, Yuichi Takei, Hidetoshi Takahashi, Yuu Kaneko, Yosuke Kita, Naruhito Hironaga, Shozo Tobimatsu, Yoko Kamio, Takashi Hanakawa, Masumi Inagaki, Kazuo Hiraki

Supporting materials

Supplementary Discussion

Temporal pole parcellation

The brain region showing sustained activation in our task was the temporal pole (the TP). The cytoarchitecture and functional connectivity of the TP had remained unknown for a long time, which was one of the reasons this area has been described as "enigmatic" [1]. However, recent studies have applied fMRI, DTI, and immunochemical detection of neurochemical markers to gradually reveal the functional-anatomical organization of the TP [2-7]. The results of these studies indicate that the TP should be divided into multiple subregions. For instance, after investigating the resting state fMRI data from 172 participants, Pascual [7] proposed that the TP should contain four major subregions forming parts of: 1) the auditory/somatosensory network, 2) the visual network, 3) the paralimbic network, and, 4) the default-semantic network. Based on their studies, we have defined the TP as regions of interest (ROI) extending from the frontal tip of the anterior temporal lobe posteriorly to the frontal part of the limen insulae (Fig. 2). We manually traced these regions, and then divided the TP into 3 regions: 1) the somatosensory network, Area TA (45 vertices, 15.27 cm^2), 2) the visual network, Areas 35 and 36 (40 vertices, 11.93 cm^2), and, 3) the semantic network, Areas TG and TE (50 vertices, 16.86 cm^2 and 21.23 cm^2, respectively). It must be noted that the paralimbic network was excluded from our analysis so that we could focus on audio-visual speech-binding mechanisms. Area TA is denoted in green in the figure and is a

part of the auditory-somatosensory network. Anteriorly, Area TA occupies the front part of the superior temporal gyrus (STG), and extends laterally from the bottom of the superior temporal sulcus, and medially to the boundary between the STG and the planum polare of the STG. Areas 35 and 36 are denoted in blue, and comprise parts of the visual network. These areas unfold anteriorly from the front part of the parahippocampal gyrus of the medial occipitotemporal gyrus, to the ventral bank of the occipitotemporal sulcus. In our study, we merged these two areas and analysed them as one. Finally, parts of the semantic-default network were defined as Areas TE (yellow) and TG (red). Anteriorly, Area TG stretches posteriorly from the tip of the TP to the frontal tip of the superior temporal sulcus; ventrally to the occipitotemporal sulcus; and ventromedially to the parahippocampus. Area TE extends along the middle and inferior temporal gyri, dorsally to the ventral part of Area TA, and caudally to the end of Area TA.

The cytoarchitecture of the temporal pole

We are particularly interested in Areas TAr and TG in the left hemisphere, which showed the strongest PAC. Based on its cytoarchitectural features and its thick and distinct layer 4 [4], TAr is classified as a granular cortical region. Compared with the other subregions in the TP such as Area TG, this area has strong parvalbumin (PV) labelling of fibres and terminals in layer 4 and the lower part of layer 3. Similarly, the immunoreactivity of nonphosphorylated neurofilament protein (SMI-32) in this area is localized to layers 5 and 6, and wisteria floribunda agglutinin (WFA) labelling is intense and mainly located deep in layers 3 and 4. Although there are large differences between the structures of subcortical regions such as the hippocampus and cortical regions such as the TAr, previous studies have indicated that parvalbumin-positive (PV+) interneurons in the hippocampus might be critical for generating PAC [8,9]. Namely, PAC is thought to involve multiple excitatory and local inhibitory processes mediated by PV+ interneurons, and experimental and theoretical studies have proposed that stabilization by recurrent inhibition is necessary for this mechanism. Therefore, unbalanced excitatory and inhibitory neural circuits are thought to decrease PAC. In fact, Wulff et al. [9] demonstrated that

PAC is significantly reduced in genetically modified mice with ablation of synaptic inhibition in PV+ interneurons. Therefore, the PAC observed in our study might be derived from recurrent inhibition mediated by PV+ interneurons. On the other hand, our data indicated that PAC was observed in Area TG. This is a dysgranular region, in which layer 2 is not as densely packed and layer 3 is more densely packed with medium-sized pyramidal neurons. In contrast to Area TAr, TG has less dense PV+ labelling overall. However, calbindin staining (CB) was strongly expressed in this area, especially in layer 3. Interestingly, these two proteins are calcium-binding proteins (CBPs), which are involved in controlling calcium levels inside the cell, and are often expressed in defined subsets of cortical GABAergic neurons [10]. Therefore, we can assume that PAC in Areas TAr and TG involves multiple processes, including excitatory and local GABAergic inhibitory processes.

Supplementary wavelet transform

To investigate whether our baseline correction using ITI was appropriate, we conducted a supplementary baseline correction, using the “preparatory periods” in place of ITI. Fig. S2 demonstrates two results of the wavelet transform using ITI and “preparatory periods” baseline corrections, respectively. These results clearly indicated that the baseline correction using ITI worked well for our data, whereas the baseline correction using the “preparatory periods” did not. Furthermore, we found that the beta oscillations were observed from the onset of the attentional cue to the end of the movie section, as has been reported by several other studies [11-13]. The results of those studies also support our selection of the ITI baseline correction.

More importantly, to investigate whether there existed a significant difference in oscillatory activities between the “preparatory periods” and “movie” segments in Fig.1B, we used t-tests to compare the z-scores in each frequency band between the two segments. We found that only delta band z-scores were significantly modulated between the periods (the movie vs. preparatory periods) in all three ROIs (the left Area TG and TAr; $t = -5.1451$, $p < 0.001$, the left Area 35,36; $t = -5.6527$, $p < 0.001$, the right Area 35, 36; $t = -4.4890$, $p < 0.001$). Bonferroni’s correction for multiple comparisons was also applied. These results suggest that the delta band activity was the

movie-related oscillatory frequency in our task.

Supplementary Figure 1.

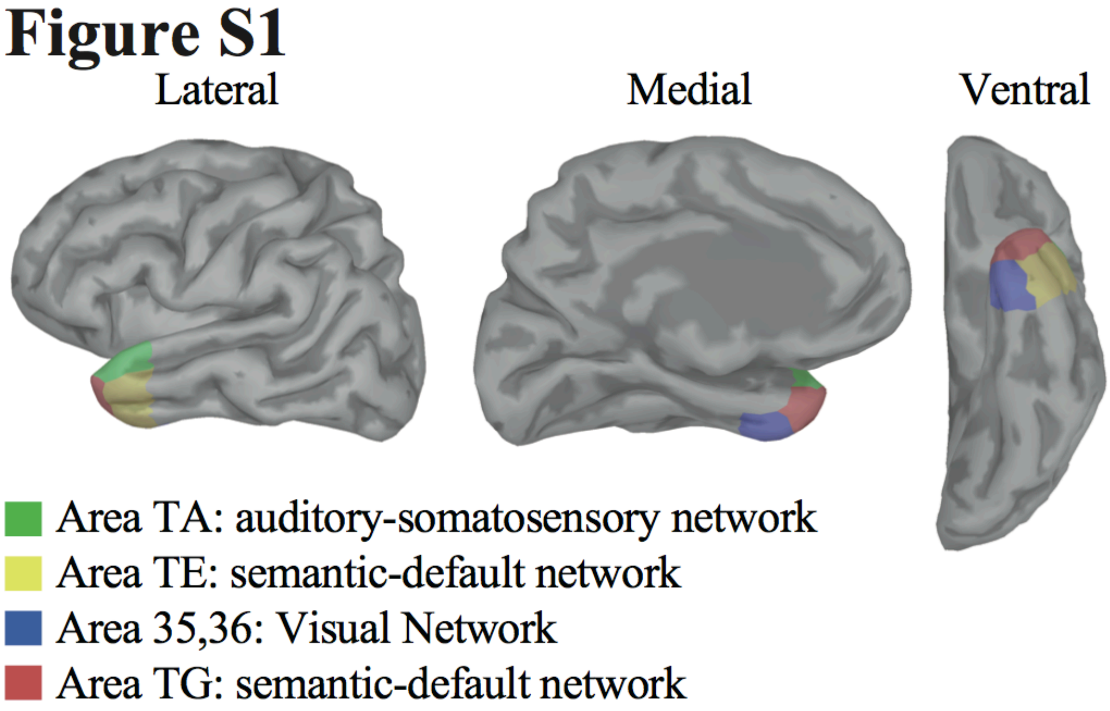

Parcellation of the left anterior temporal lobe (TP). The coloured parcellation map contains 4 sub-regions of the TP: Area TA, TE, 35 and 36, and area TG. This area is considered a convergent hub region, so we parcelled this region into four subregions as per a previous study (Pascual et al. 2015).

Supplementary Figure 2.

Figure S2

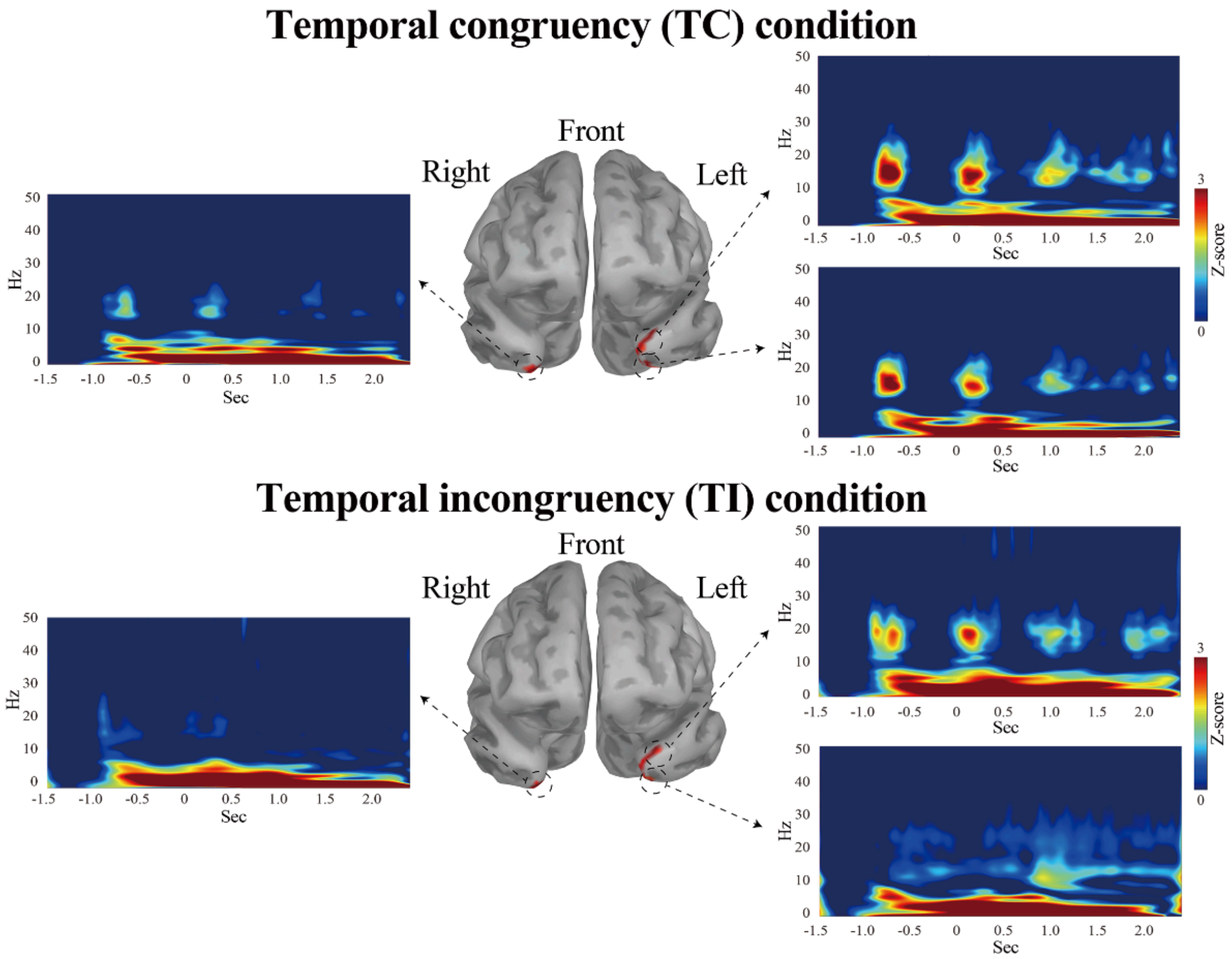

Z-score of wavelet transformation of source data from three brain areas (ROIs) in the TP. As a supplementary analysis, a whole data segment from the attentional cue, the fixation point, and the movie was analysed, using wavelet transform (3900 ms). As clearly seen, the delta and beta bands oscillations demonstrated remarkable traits. Then, we compared z-scores of the wavelet transform of our source data in three brain areas (ROIs) in the TP between the pre-stimulus periods and the movie section. It can be clearly seen that the delta rhythms were significantly modulated between the movie segment and the preparatory periods, including the attentional cue and the fixation point (the left Area TG and TAr, the left Area 35, 36, and the right Area 35, 36, Bonferroni correction, $p < 0.001$). This result indicates that the delta oscillations were movie (audio-visual)-related oscillations in our task. Interestingly, we also observed oscillatory activities in beta bands from the preparatory periods to the movie. In this measurement, we used

the signal in the last 500 ms of ITI as the baseline. The horizontal line denotes time sequence, and the vertical line indicates frequency (Hz). Upper figure; the TC condition, Lower figure; the TI condition.

PAC and binding of audio and visual components of speech in the TP

One intriguing difference between our data from the TP and other PAC studies was found: The frequency band coupling differences. We found delta-beta coupling, whereas the previous studies cited above reported theta-gamma coupling. We hypothesize that the difference lies in species differences in oscillatory frequencies. That is, humans have the slowest frequencies of all investigated species. For example, hippocampal theta oscillations typically range from 5-8 Hz in humans, whereas hippocampal theta ranges from 6-10 Hz in rodents. This applies to fast activity as well: The "ripple" oscillation is 110 Hz in humans, but is 130-160 Hz in rats. A potential mechanism underlying the decreased frequencies in humans is axonal conduction delays related to the considerably greater human brain size [14]. This would be consistent with our findings in the TP, especially since the TP is a convergent hub region that, when measured by diffusion tensor imaging, appears to require the longest axonal lengths in the brain [2,5].

Beta band frequencies represent top-down processing

We interpreted PAC in the TP as a neural binding mechanism for information from different sensory cortices. However, some current studies have proposed that beta activity could represent top-down operations [15,16]. That view is relevant to our data, and thus warrants further discussion. According to previous studies, two functional roles of beta rhythms as top-down signals have been proposed. First, beta oscillations are mostly believed to predict only timing, and enhance other mechanisms to process stimuli; and second, a more controversial suggestion is that beta frequencies encode both timing and content [12,17-19]. Beta oscillations in our data were observed

from the onset of the attentional cue to the end of the movie section (Fig. S1), similarly to results obtained elsewhere [11-13]. Thus, we speculated that the beta band oscillations of the TP provide multi-sensory feedback to the primary cortices, and through this the TP modulates or directs information processed in the unisensory cortex. These phenomena can be described as “active sensing”, whereby we actively sample and process the information with a rhythmic mode implemented by neuronal oscillations. Some previous studies have demonstrated that beta oscillations in motor systems play a pivotal role in active sensing, which involves cooperation between motor and sensory systems to enhance information processing. For instance, an observer’s experience of a rhythmic sound affects not only the auditory cortex, but also the motor cortex [19]. Thus, the beta rhythms in the TP might be partly explained by the active-sensing paradigm. However, our subsequent coherence measurements of the beta oscillations do not support the idea that TP-sourced beta oscillations interacted with and modulated activity in other brain regions (e.g., primary sensory areas). Because of these conflicting results, this issue remains elusive. Therefore, future studies are expected to answer the question of the functional roles of beta activities in the multisensory cortices more directly.

Supplementary References

1 Olson, I. R., Plotzker, A. & Ezzyat, Y. The Enigmatic temporal pole: a review of findings on social and emotional processing. *Brain.* **130**, 1718-1731, doi:10.1093/brain/awm052 (2007).

2 Binney, R. J., Parker, G. J. & Lambon Ralph, M. A. Convergent connectivity and graded specialization in the rostral human temporal lobe as revealed by diffusion-weighted imaging probabilistic tractography. *J. Cogn. Neurosci.* **24**, 1998-2014, doi:10.1162/jocn_a_00263 (2012).

3 Blaizot, X. *et al.* The human parahippocampal region: I. Temporal pole cytoarchitectonic

and MRI correlation. *Cereb. Cortex.* **20**, 2198-2212, doi:10.1093/cercor/bhp289 (2010).

4 Ding, S. L., Van Hoesen, G. W., Cassell, M. D. & Poremba, A. Parcellation of human temporal polar cortex: a combined analysis of multiple cytoarchitectonic, chemoarchitectonic, and pathological markers. *J. Comp. Neurol.* **514**, 595-623, doi:10.1002/cne.22053 (2009).

5 Fan, L. *et al.* Connectivity-based parcellation of the human temporal pole using diffusion tensor imaging. *Cereb. Cortex.* **24**, 3365-3378, doi:10.1093/cercor/bht196 (2014).

6 Insausti, R. *et al.* MR volumetric analysis of the human entorhinal, perirhinal, and temporopolar cortices. *AJNR Am. J. Neuroradiol.* **19**, 659-671 (1998).

7 Pascual, B. *et al.* Large-scale brain networks of the human left temporal pole: a functional connectivity MRI study. *Cereb. Cortex.* **25**, 680-702, doi:10.1093/cercor/bht260 (2015).

8 Buzsaki, G. & Watson, B. O. Brain rhythms and neural syntax: implications for efficient coding of cognitive content and neuropsychiatric disease. *Dialogues. Clin. Neurosci.* **14**, 345-367 (2012).

9 Wulff, P. *et al.* Hippocampal theta rhythm and its coupling with gamma oscillations require fast inhibition onto parvalbumin-positive interneurons. *Proc. Natl. Acad. Sci. U S A.* **106**, 3561-3566, doi:10.1073/pnas.0813176106 (2009).

10 Kawaguchi, Y. & Kubota, Y. GABAergic cell subtypes and their synaptic connections in rat frontal cortex. *Cereb. Cortex.* **7**, 476-486 (1997).

11 Arnal, L. H., Doelling, K. B. & Poeppel, D. Delta-Beta Coupled Oscillations Underlie Temporal Prediction Accuracy. *Cereb. Cortex.* **25**, 3077-3085, doi:10.1093/cercor/bhu103 (2015).

12 Doelling, K. B. & Poeppel, D. Cortical entrainment to music and its modulation by expertise. *Proc. Natl. Acad. Sci. U S A.* **112**, E6233-6242, doi:10.1073/pnas.1508431112 (2015).

13 Senkowski, D., Schneider, T. R., Foxe, J. J. & Engel, A. K. Crossmodal binding through neural coherence: implications for multisensory processing. *Trends Neurosci.* **31**, 401-409, doi:10.1016/j.tins.2008.05.002 (2008).

14 Buzsaki, G., Logothetis, N. & Singer, W. Scaling brain size, keeping timing: evolutionary preservation of brain rhythms. *Neuron.* **80**, 751-764, doi:10.1016/j.neuron.2013.10.002 (2013).

15 Bastos, A. M. *et al.* Visual areas exert feedforward and feedback influences through distinct frequency channels. *Neuron.* **85**, 390-401, doi:10.1016/j.neuron.2014.12.018 (2015).

16 Buschman, T. J. & Miller, E. K. Top-down versus bottom-up control of attention in the prefrontal and posterior parietal cortices. *Science.* **315**, 1860-1862, doi:10.1126/science.1138071 (2007).

17 Arnal, L. H. Predicting "When" Using the Motor System's Beta-Band Oscillations. *Front. Hum. Neurosci.* **6**, 225, doi:10.3389/fnhum.2012.00225 (2012).

18 Arnal, L. H. & Giraud, A. L. Cortical oscillations and sensory predictions. *Trends Cogn. Sci.* **16**, 390-398, doi:10.1016/j.tics.2012.05.003 (2012).

19 Fujioka, T., Trainor, L. J., Large, E. W. & Ross, B. Internalized timing of isochronous sounds is represented in neuromagnetic beta oscillations. *J. Neurosci.* **32**, 1791-1802, doi:10.1523/JNEUROSCI.4107-11.2012 (2012).
